# Crystal chemical design, synthesis and characterisation of U(IV)-dominant betafite phases for actinide immobilisation

**DOI:** 10.1038/s41598-023-36571-w

**Published:** 2023-06-26

**Authors:** Shi-Kuan Sun, Lucy M. Mottram, Thomas Gouder, Martin C. Stennett, Neil C. Hyatt, Claire L. Corkhill

**Affiliations:** 1 Immobilisation Science Laboratory, Department of Materials Science and Engineering, University of Sheffield, Sheffield, S1 3JD UK; 2 School of Material Science and Energy Engineering, Foshan University, Foshan, 528000 Guangdong China; 3 European Commission, Joint Research Centre (JRC), Postfach 2340, 76125 Karlsruhe, Germany; 4 School of Mechanical and Materials Engineering, Washington State University, Pullman, WA 99164 USA; 5 School of Earth Sciences, University of Bristol, Bristol, BS8 1RJ UK

**Keywords:** Ceramics, Solid-state chemistry

## Abstract

Crystal chemical design principles were applied to synthesise novel U^4+^ dominant and titanium excess betafite phases Ca_1.15(5)_U_0.56(4)_Zr_0.17(2)_Ti_2.19(2)_O_7_ and Ca_1.10(4)_U_0.68(4)_Zr_0.15(3)_Ti_2.12(2)_O_7_, in high yield (85–95 wt%), and ceramic density reaching 99% of theoretical. Substitution of Ti on the A-site of the pyrochlore structure, in excess of full B-site occupancy, enabled the radius ratio (r_A_/r_B_ = 1.69) to be tuned into the pyrochlore stability field, approximately 1.48 ≲ r_A_/r_B_ ≲ 1.78, in contrast to the archetype composition CaUTi_2_O_7_ (r_A_/r_B_ = 1.75). U L_3_-edge XANES and U *4f*_7/2_ and U *4f*_5/2_ XPS data evidenced U^4+^ as the dominant speciation, consistent with the determined chemical compositions. The new betafite phases, and further analysis reported herein, point to a wider family of actinide betafite pyrochlores that could be stabilised by application of the underlying crystal chemical principle applied here.

## Introduction

Ceramic materials are considered as leading candidate wasteforms for the immobilisation and geological disposal of long lived actinides arising from nuclear fuel cycles and medical radioisotope production^1–7^. Titanate ceramics with the pyrochlore structure are of particular interest for such applications, given the long term stability of natural mineral analogues with substantial uranium or thorium inventory^1,2,4,8,9^. Pyrochlore-group minerals with the prototypical formula of A_2-m_B_2_O_6_(O,OH,F)_1−n_, comprise three subgroups (pyrochlore, microlite and betafite) classified according to B-site composition; the betafite subgroup is defined as having 2 Ti_B_ ≥ (Nb + Ta)_B_^10^. Naturally occurring minerals of the pyrochlore group have been shown to be stable under environmental conditions retaining actinides effectively over geological time periods, in excess of 1 billion years, much longer than the performance period of a geological disposal facility^11–13^.

Taking the simplified formula A_2_B_2_O_6_O’, the pyrochlore structure may be described as interpenetrating B_2_O_6_ and (anti-cristobalite) A_2_O’ networks, with corner sharing BO_6_ octahedra and distorted AO_8_ scalenohedra. The pyrochlore structure is related to the fluorite structure (comparable formula A_2_B_2_O_8_), by ordering of both cations and oxygen vacancies, leading to a 2 × 2 × 2 superstructure, relative to the fluorite unit cell (a_p_ = 2 a_f_). The pyrochlore structure is stabilised, under ambient conditions, within the approximate radius ratio range: 1.46 ≲ r_A_/r_B_ ≲ 1.78: below this threshold, a defect fluorite phase is stabilised, with cation and oxygen vacancy disorder; whereas, above the threshold, a monoclinic structure is stabilised, typified by La_2_Ti_2_O_7_^8,14^.

The archetype betafite CaUTi_2_O_7_ is of specific interest as ceramic wasteform for actinide disposition, it is a component of the multiphase Synroc F wasteform and a ceramic phase assemblage designed to immobilise U-rich waste from ^99^Tc production^4,15–19^. There is a consensus that solid state synthesis of near single phase CaUTi_2_O_7_, with ≳ 95 wt% yield, is problematic^16–19^. Dickson et al*.* noted that CaUTi_2_O_7_ “*invariably coexisted with substantial portions of perovskite (CaTiO*_*3*_*) and uraninite (UO*_*2*_*)*”^16^; and Vance et al*.* reported “*several days failed to assure complete reaction… and the pyrochlore yields did not exceed* ~ *75 wt%*”^18^. These results are perhaps not altogether surprising given, that the radius ratio of CaUTi_2_O_7_, r_A_/r_B_ = 1.75, is on the cusp of the pyrochlore stability field (herein, Shannon’s effective ionic radii are employed^20^). Interestingly, the radius ratio may be tuned into the stability field of the pyrochlore structure by oxidation of U^4+^ to U^5+^/U^6+^, with coupled charge substitution, for example: Ca_1.4_U_0.7_Ti_2_O_7_, with U^4.5+^ and r_A_/r_B_ = 1.71^21^. However, U^4+^ is the preferred speciation for wasteform applications, given the lower solubility and compatibility with reducing groundwaters, expected at depth, in a geological disposal facility. Vanderah et al*.* established that the pyrochlore structure may be stabilised for relatively large A-site cations, by substitution on the A-site of typical B-site cations, in excess of full B-site occupancy; remarkably, up to 25% substitution on the A-site may be tolerated^22^. We therefore applied this crystal chemical design principle to hypothesise novel U^4+^ dominant, and titanium excess, betafite compositions with radius ratio, r_A_/r_B_ = 1.69, within the pyrochlore stability field, nominally Ca_1.00_U_0.50_Zr_0.20_Ti_2.30_O_7_ and Ca_0.96_U_0.72_Zr_0.17_Ti_2.15_O_7_. Herein, we report the successful synthesis and characterisation of such betafite compounds. Our hypothesis was guided by the observation of a nominally B-site stoichiometric pyrochlore phase by Vance et al*.* in the zirconolite solid solution of CaZr_1−x_U_x_Ti_2_O_7_ with x = 0.7, apparently co-existing with a zirconolite 4M phase^18^. The novel betafite phases designed and reported herein, point to a wider family of actinide pyrochlores that could be stabilised by application of the same crystal chemical principle, which we hope will be more extensively investigated.

## Results and discussion

**Figure 1:**
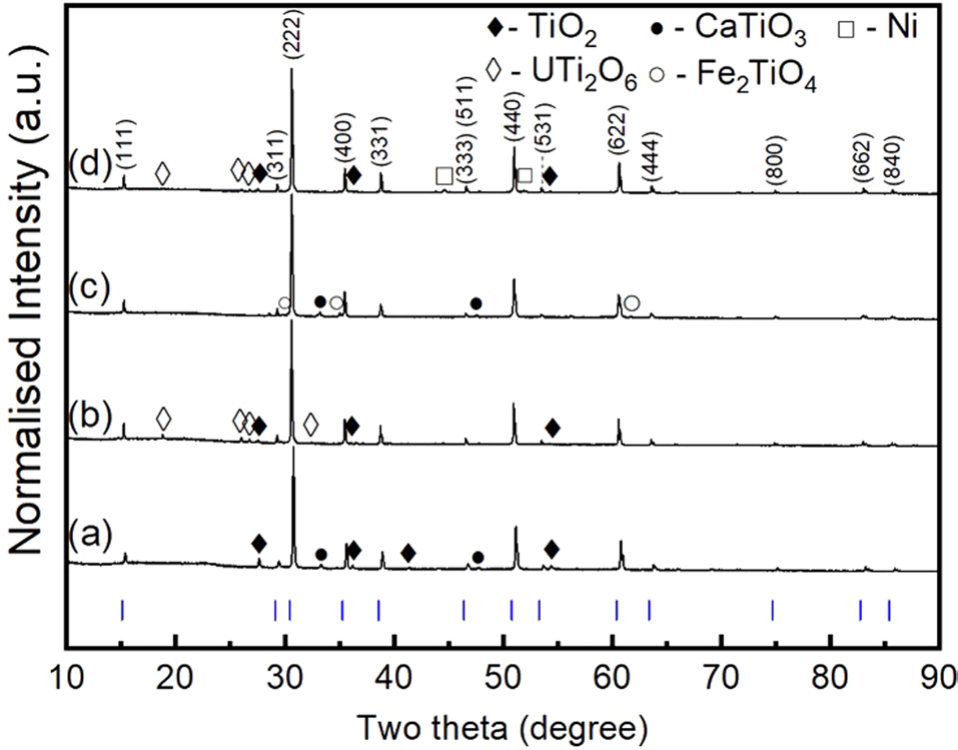
PXRD patterns of the product after sintering of (**a**) nominal Ca_1.00_U_0.50_Zr_0.20_Ti_2.30_O_7_, (**b**) nominal Ca_0.96_U_0.72_Zr_0.17_Ti_2.15_O_7_, and (**c**) 10wt% Fe addition to nominal Ca_0.96_U_0.72_Zr_0.17_Ti_2.15_O_7_ (**d**) 10wt% Ni addition to nominal Ca_0.96_U_0.72_Zr_0.17_Ti_2.15_O_7_; compounds were synthesised at 1320 °C in flowing N_2_. Vertical marks are Bragg reflections determined from the reported structure of Ca_0.92_U_1.08_Ti_1.99_O_7_^16^.

Powder X-ray diffraction (PXRD) analysis of the synthesised products demonstrated the formation of pyrochlore structured compounds (space group *F*d $\overline{3 }$ m) with the presence of only minor or trace secondary phases (Fig. 1). The clear presence of (111) and (311) reflections, indexed in *F*d $\overline{3 }$ m, at 2θ ≈ 15° and 2θ ≈ 30°, respectively, was diagnostic of cation and oxygen vacancy ordering, characteristic of a pyrochlore structure. The calculated lattice parameters of nominal Ca_1.00_U_0.50_Zr_0.20_Ti_2.30_O_7_ and Ca_0.96_U_0.72_Zr_0.17_Ti_2.15_O_7_, *a* = 10.1215(2) Å and *a* = 10.1374(1) Å, respectively, were slightly smaller than those of the previously reported betafite phase, Ca_0.92_U_1.08_Ti_1.99_O_7_, *a* = 10.1579 Å^16^. This is in accordance with the comparably greater radius ratio of Ca_0.92_U_1.08_Ti_1.99_O_7_, r_A_/r_B_ = 1.74.

For the nominal Ca_1.00_U_0.50_Zr_0.20_Ti_2.30_O_7_ composition, TiO_2_ (rutile) and CaTiO_3_ (perovskite) were detected as the minor phases, as shown in Fig. 1a. Whereas, for the nominal Ca_0.96_U_0.72_Zr_0.17_Ti_2.15_O_7_ content, only trace impurities of TiO_2_ and UTi_2_O_6_ (brannerite) were observed, as shown Fig. 1b. These data imply that an intermediate composition between these end members should yield a truly single phase material. Addition of 10 wt%. Fe and Ni was made to the target composition Ca_0.96_U_0.72_Zr_0.17_Ti_2.15_O_7_, for the purpose of scavenging potential trace oxygen from the nitrogen gas atmosphere used in synthesis (see "Methods" section). Addition of 10 wt.% Fe to nominal composition Ca_0.96_U_0.72_Zr_0.17_Ti_2.15_O_7_, led to the formation of Fe_2_TiO_4_ (ulvospinel) in addition to CaTiO_3_, as shown in Fig. 1c. Addition of 10 wt.% Ni to nominal composition Ca_0.96_U_0.72_Zr_0.17_Ti_2.15_O_7_, was found to not influence the phase assemblage, and unreacted Ni metal was retained, Fig. 1d. No free uranium oxides were detected in the XRD of any product, which, together with the well sintered microstructures (see below), suggested that the solid state reactions were not kinetically hindered.

**Figure 2:**
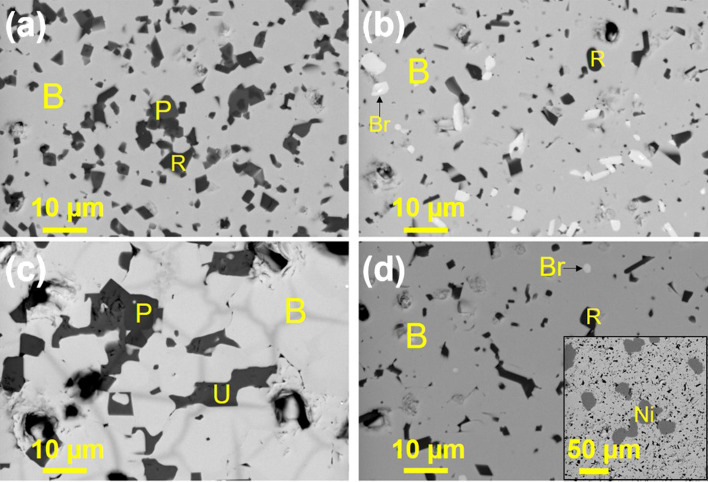
Scanning Electron Microscopy (SEM) observation in backscattered electron mode of the polished surface (**a**) nominal Ca_1.00_U_0.50_Zr_0.20_Ti_2.30_O_7_, (**b**) nominal Ca_0.96_U_0.72_Zr_0.17_Ti_2.15_O_7_, and (**c**) 10wt% Fe addition to nominal Ca_0.96_U_0.72_Zr_0.17_Ti_2.15_O_7_ (**d**) 10wt% Ni addition to nominal Ca_0.96_U_0.72_Zr_0.17_Ti_2.15_O_7_. The inset of (**d**) shows the presence of Ni phase in low magnification. Labels highlight: *B* the betafite major phase, *R* rutile, *P* perovskite, *Br* brannerite, *U* ulvospinel, and *Ni* nickel.

The microstructures of sintered betafite ceramics are shown in Fig. 2, and were fully consistent with the phase assemblage determined from XRD data. The Energy Dispersive X-ray (EDX) determined compositions of constituent phases are presented in Table 1 and Table S1*(supplementary material)*; EDX spectra are presented in Figs. S1–S4*.*

**Table 1:** The EDX quantitative analysis of betafite phases (7 O atoms per formula unit assumed).

Target composition	Cation stoichiometry of betafite phase (f.u.)				
	Ca	U	Zr	Ti	Fe
Ca_1.00_U_0.50_Zr_0.20_Ti_2.30_O_7_	1.15 (5)	0.56 (4)	0.17 (2)	2.19 (2)	–
Ca_0.96_U_0.72_Zr_0.17_Ti_2.15_O_7_	1.10 (4)	0.68 (4)	0.15 (3)	2.12 (2)	–
Ca_0.96_U_0.72_Zr_0.17_Ti_2.15_O_7_ + 10wt% Fe	0.90 (5)	0.71 (5)	0.15 (2)	1.97 (3)	0.28(6)
Ca_0.96_U_0.72_Zr_0.17_Ti_2.15_O_7_ + 10wt.% Ni	1.03 (2)	0.64 (3)	0.14 (2)	2.17 (3)	–

Nominal composition Ca_1.00_U_0.50_Zr_0.20_Ti_2.30_O_7_ exhibited a dense microstructure, with little porosity observed (Fig. 2a). From greyscale contrast, it was evident that the microstructure comprised three distinct phases. The major phase (labelled B) was identified as betafite, as determined by the coincidence of U, Ca and Ti signals in EDX spectra (Fig. S1). The presence of the Zr Lα emission line at *ca.* 2 keV was indicative of the solid solution of Zr in the betafite phase of all products. Minor phases were determined to be TiO_2_ and CaTiO_3_ (labelled R and P, respectively; EDX spectra presented in Fig. S1). Nominal composition Ca_0.96_U_0.72_Zr_0.17_Ti_2.15_O_7_ also presented a dense microstructure, Fig. 2b, that comprised a majority betafite phase with minor TiO_2_ and UTi_2_O_6_ (labelled Br; EDX spectra presented in Fig. S2). The addition of 10wt% Fe was found to have a significant impact on the phase assemblage and microstructure of nominal composition Ca_0.96_U_0.72_Zr_0.17_Ti_2.15_O_7_. As shown in Fig. 2c, in addition to a major betafite phase, minor CaTiO_3_ and Fe_2_TiO_4_ (ulvospinel, labelled U) were observed, together with considerable porosity (EDX spectra presented in Fig. S3). 10wt% Ni addition to nominal composition Ca_0.96_U_0.72_Zr_0.17_Ti_2.15_O_7_ also produced a dense microstructure, Fig. 2d, that comprised a majority betafite phase with minor TiO_2_ and UTi_2_O_6_, and residual Ni metal (EDX spectra presented in Fig. S4). The observed size of the Ni phase (see inset to Fig. 2d) was consistent with that of the starting Ni metal reagent. This, and the absence of any Ni Kα emission line in the EDX spectra of Fig. S4a–c, demonstrated no detectable reaction of the Ni metal had occurred.

The EDX chemical compositions of the major betafite phases were close to those targeted and evidenced an excess of B-site cations within precision: Ca_1.15(5)_U_0.56(4)_Zr_0.17(2)_Ti_2.19(2)_O_7_ and Ca_1.10(4)_U_0.68(4)_Zr_0.15(3)_Ti_2.12(2)_O_7_, for nominal Ca_1.00_U_0.50_Zr_0.20_Ti_2.30_O_7_ and Ca_0.96_U_0.72_Zr_0.17_Ti_2.15_O_7_, respectively (see Table 1). The EDX determined compositions implied average uranium oxidation states of 4.04 + and 4.00 +, respectively, assuming Ti^4+^ speciation (note: synthesis conditions were not considered sufficiently reducing to afford significant reduction to Ti^3+^). The composition of the betafite phase in nominal Ca_0.96_U_0.72_Zr_0.17_Ti_2.15_O_7_ with 10 wt.% Fe addition was Ca_0.90(5)_U_0.71(5)_Zr_0.15(2)_Ti_1.97(3)_Fe_0.28(6)_O_7_. Evidently, Fe was incorporated into the crystal structure; assuming speciation as Fe^3+^, the composition implied an average uranium oxidation state of 4.05 +. The chemical composition of the betafite phase in nominal Ca_0.96_U_0.72_Zr_0.17_Ti_2.15_O_7_ with 10wt.% Ni addition, was determined to be Ca_1.03(2)_U_0.64(3)_Zr_0.14(2)_Ti_2.17(3)_O_7_, with an implied uranium oxidation state of 4.21 + . The EDX determined composition was close to that of the Ni free counterpart composition.

Rietveld analysis of PXRD data was performed to allow quantitative phase analysis (QPA) of the products and the results are summarised in Table 2; target compositions were used as the structural model. The largest fraction of betafite was found for the nominal Ca_1.00_U_0.50_Zr_0.20_Ti_2.30_O_7_ composition: betafite—94.58 wt.%, rutile—2.14 wt.%, brannerite—3.29 wt.%; for other compositions, the betafite phase comprised approximately 85 wt.% of the phase assemblage. Combining QPA, EDX analyses, and measured bulk densities, it was possible to estimate the relative density of the ceramic materials, which are presented in Table S2.

**Table 2:** Phase assemblage derived from Rietveld analysis of PXRD data; EDX compositions of betafite and secondary phases are reported, respectively, in Table 1 and Table S1.

Composition	Weight fraction (wt.%)						R_wp_ (%)	χ^2^
	Betafite	CaTiO_3_	TiO_2_	UTi_2_O_6_	Fe_2_TiO_4_	Ni		
Ca_1.00_U_0.50_Zr_0.20_Ti_2.30_O_7_	84.80 ± 0.24%	4.56 ± 0.59	10.64 ± 0.65	–	–	–	9.77	2.99
Ca_0.96_U_0.72_Zr_0.17_Ti_2.15_O_7_	94.58 ± 0.07%	–	2.14 ± 1.20	3.29 ± 0.86	–	–	8.75	3.86
Ca_0.96_U_0.72_Zr_0.17_Ti_2.15_O_7_ + 10wt% Fe	86.42 ± 0.11%	6.78 ± 0.66	–	–	6.80 ± 1.02	–	6.82	2.92
Ca_0.96_U_0.72_Zr_0.17_Ti_2.15_O_7_ + 10wt.% Ni	85.82 ± 0.11%	–	3.74 ± 0.63	5.68 ± 0.46	–	4.76 ± 0.46	8.72	4.43

**Figure 3:**
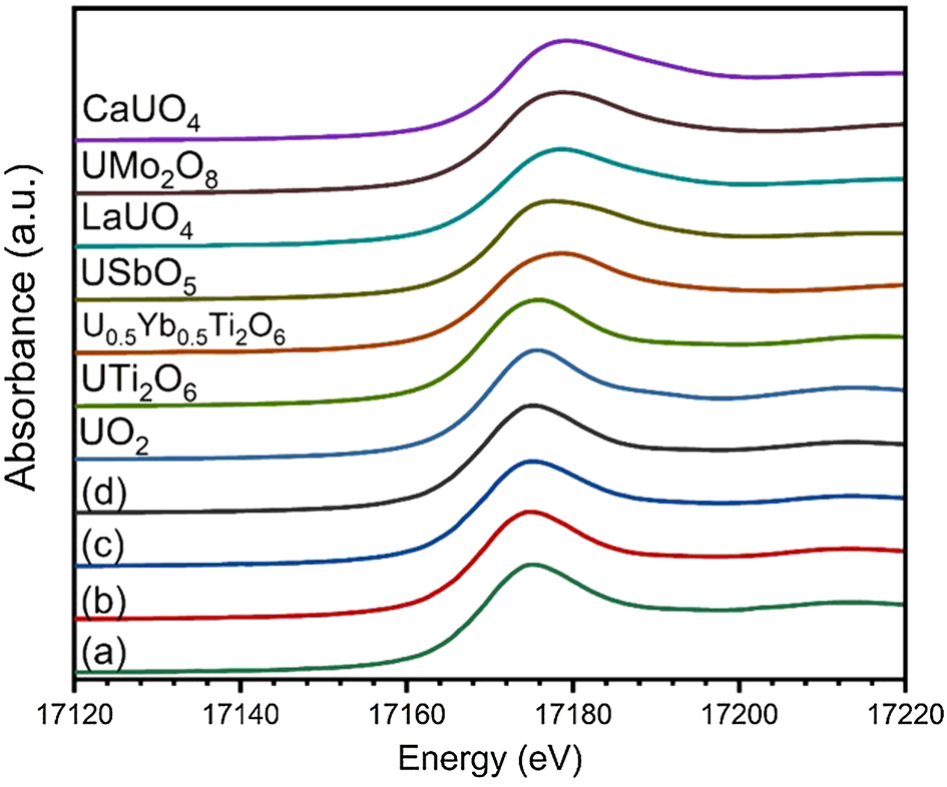
U L_3_-edge XANES data of (**a**) nominal Ca_1.00_U_0.50_Zr_0.20_Ti_2.30_O_7_, (**b**) nominal Ca_0.96_U_0.72_Zr_0.17_Ti_2.15_O_7_, and (**c**) 10wt.% Fe addition to nominal Ca_0.96_U_0.72_Zr_0.17_Ti_2.15_O_7_ composition (**d**) 10wt.% Ni addition to nominal Ca_0.96_U_0.72_Zr_0.17_Ti_2.15_O_7_ composition. Also shown are spectra of reference compounds: U^4+^ in UO_2_, UTi_2_O_6_; U^5+^ in U_0.5_Y_0.5_Ti_2_O_6_, USbO_5_, LaUO_4_, UMo_2_O_8_; and U^6+^ in CaUO_4_.

**Figure 4:**
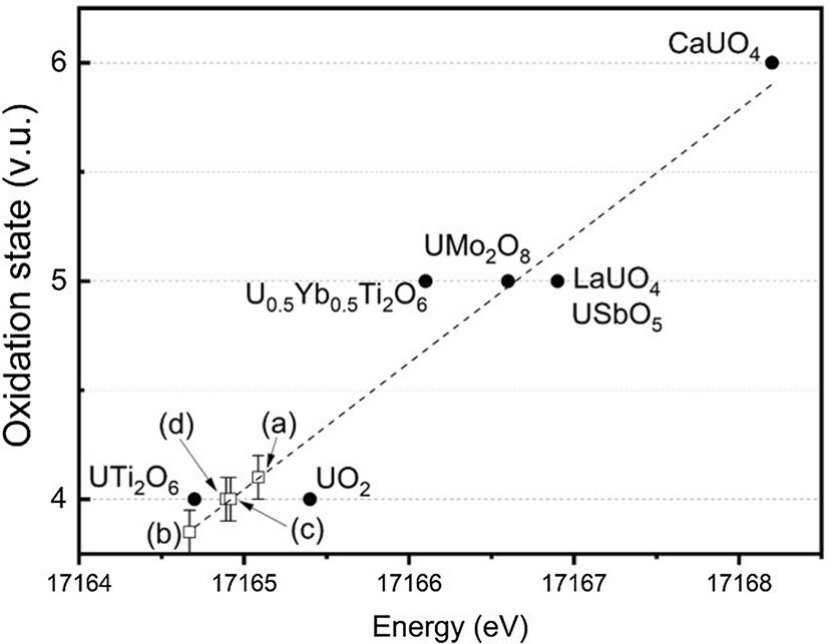
The oxidation state as a function of edge position (E_0_) for uranium compounds together with a model linear fit (the dashed line; R^2^ = 0.913). Data points correspond to (**a**) nominal Ca_1.00_U_0.50_Zr_0.20_Ti_2.30_O_7_, (**b**) nominal Ca_0.96_U_0.72_Zr_0.17_Ti_2.15_O_7_. Reference compounds represent: U^4+^ in UO_2_, UTi_2_O_6_; U^5+^ in U_0.5_Y_0.5_Ti_2_O_6_, USbO_5_, LaUO_4_, UMo_2_O_8_; and U^6+^ in CaUO_4_.

The nominal Ca_1.00_U_0.50_Zr_0.20_Ti_2.30_O_7_ and Ca_0.96_U_0.72_Zr_0.17_Ti_2.15_O_7_ ceramics were estimated to have a relative density of approximately 99% of theoretical, whereas the estimated relative densities of the ceramics with 10wt.% Fe and 10wt.% Ni were somewhat lower. This was consistent with the observation or absence of porosity in the corresponding microstructures shown in Fig. 2 and discussed above. The average bulk uranium oxidation state in the betafite ceramics was investigated by analysis of X-ray absorption near edge structure (XANES) at the U L_3_-edge; data are presented in Fig. 3. The oxidation state was determined by the linear regression method as has been previously proposed^23,24^, using the edge position, E_0_, of reference compounds of known oxidation state to establish a calibration line, as shown in Fig. 4. The bulk average uranium oxidation states determined by this method evidenced the presence of only U^4+^ in the betafite ceramics, within experimental error, as shown in Table 3). The difference in features of the white line maximum and near-edge structure of the U L_3_-XANES, for compounds with the same nominal oxidation state shown in Fig. 3, reflect sensitivity to the specific local environment of the U absorber in the reference compounds.

XANES data were also analysed by combinatorial linear combination fitting (LCF)^25,26^, using the library of reference compounds to estimate the fraction of contributing oxidation states. Significance tests of the goodness of fit R-factor were undertaken using the Hamilton *R*-factor ratio test, with a significance level of *α* = 0.05^27^. From these fits the weighted mean oxidation state and associated root mean square error approximation were calculated. The plots of best fit are shown in Fig. S5. The results of the combinatorial LCF, summarised in Table 3, also evidenced a dominant average bulk uranium oxidation state of U^4+^, but with a minor U^5+^ contribution; no significant U^6+^ contribution was determined.

**Table 3:** The average oxidation state of uranium estimated from EDX analysis of the betafite phase (a) nominal Ca_1.00_U_0.50_Zr_0.20_Ti_2.30_O_7_, (b) nominal Ca_0.96_U_0.72_Zr_0.17_Ti_2.15_O_7_, (c) nominal Ca_0.96_U_0.72_Zr_0.17_Ti_2.15_O_7_ + 10wt% Fe and (d) nominal Ca_0.96_U_0.72_Zr_0.17_Ti_2.15_O_7_ + 10wt% Ni (Table 1), linear regression (LR) and combinatorial linear combination analysis (cLCF) of bulk U L_3_-edge XANES data, and XPS from deconvolution of the U 4*f*_7/2_ photoelectron peaks (*not measured).

Composition		a	b	c	d
Oxidation state	EDX	4.04	4.00	4.05	4.21
	XANES LR	4.10 ± 0.10	3.90 ± 0.10	4.00 ± 0.10	4.00 ± 0.10
	XANES cLCF	4.09 ± 0.04	4.06 ± 0.19	4.21 ± 0.11	4.05 ± 0.16
	XPS	4.09 ± 0.04	4.12 ± 0.04	*	*
Combinatorial LCF	Mean contribution to spectrum	UO_2_: 2.8%, UTi_2_O_6_: 83.4%	UTi_2_O_6_: 87.1%	UTi_2_O_6_: 76.1%	UTi_2_O_6_: 91.4%
		USbO_5_: 12.8%	LaUO_4_: 9.1%, USbO_5_: 2.3%	LaUO_4_: 5.1% USbO_5_: 18.3%	LaUO_4_: 7.9%
XPS	Contribution to spectrum	U^4+^: 91%	U^4+^: 88%	*	*
		U^5+^: 9%	U^5+^: 12%		

**Figure 5:**
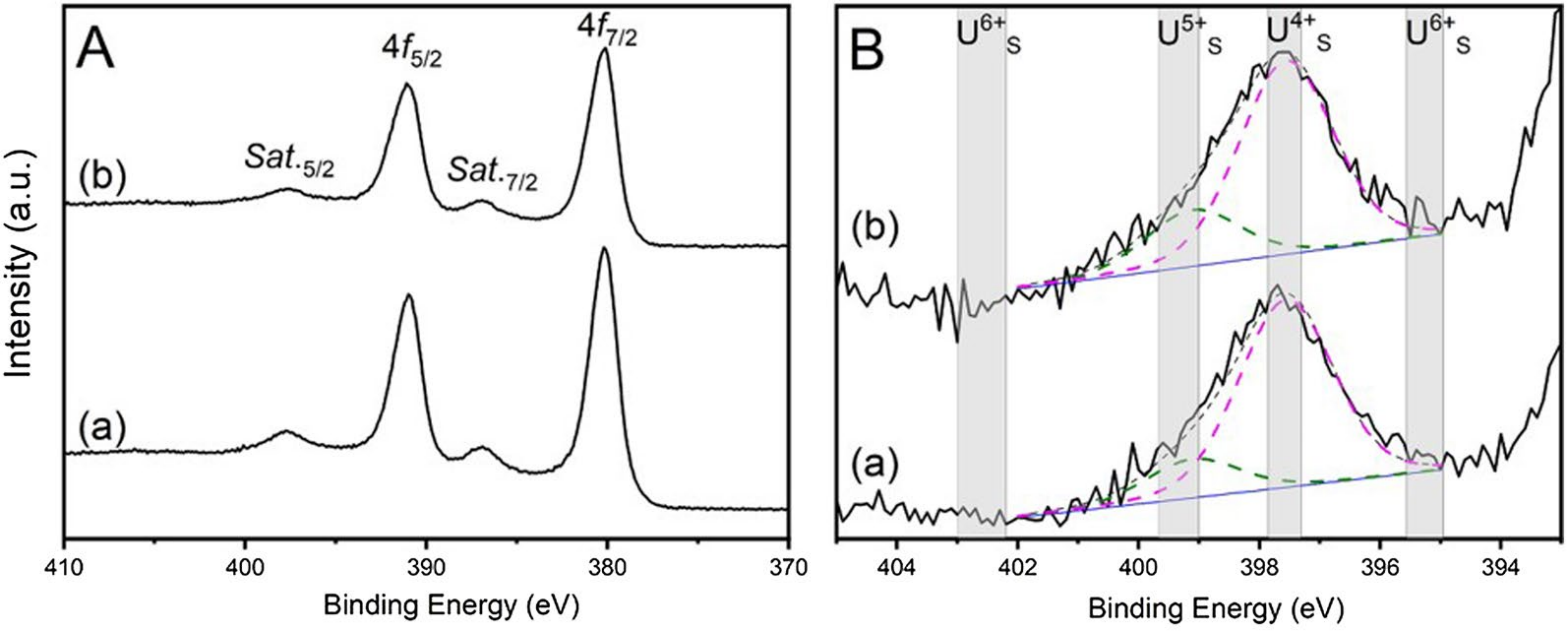
(**A**) The U *4f*_7/2_ and U *4f*_5/2_ regions of the XPS spectra for (a) nominal Ca_1.00_U_0.50_Zr_0.20_Ti_2.30_O_7_ and (b) nominal Ca_0.96_U_0.72_Zr_0.17_Ti_2.15_O_7_. (**B**) Satellite peaks in the range of 393–405 eV with the fitting of the components of U^4+^ and U^5+^ for (a) nominal Ca_1.00_U_0.50_Zr_0.20_Ti_2.30_O_7_ and (b) nominal Ca_0.96_U_0.72_Zr_0.17_Ti_2.15_O_7_.

**Figure 6:**
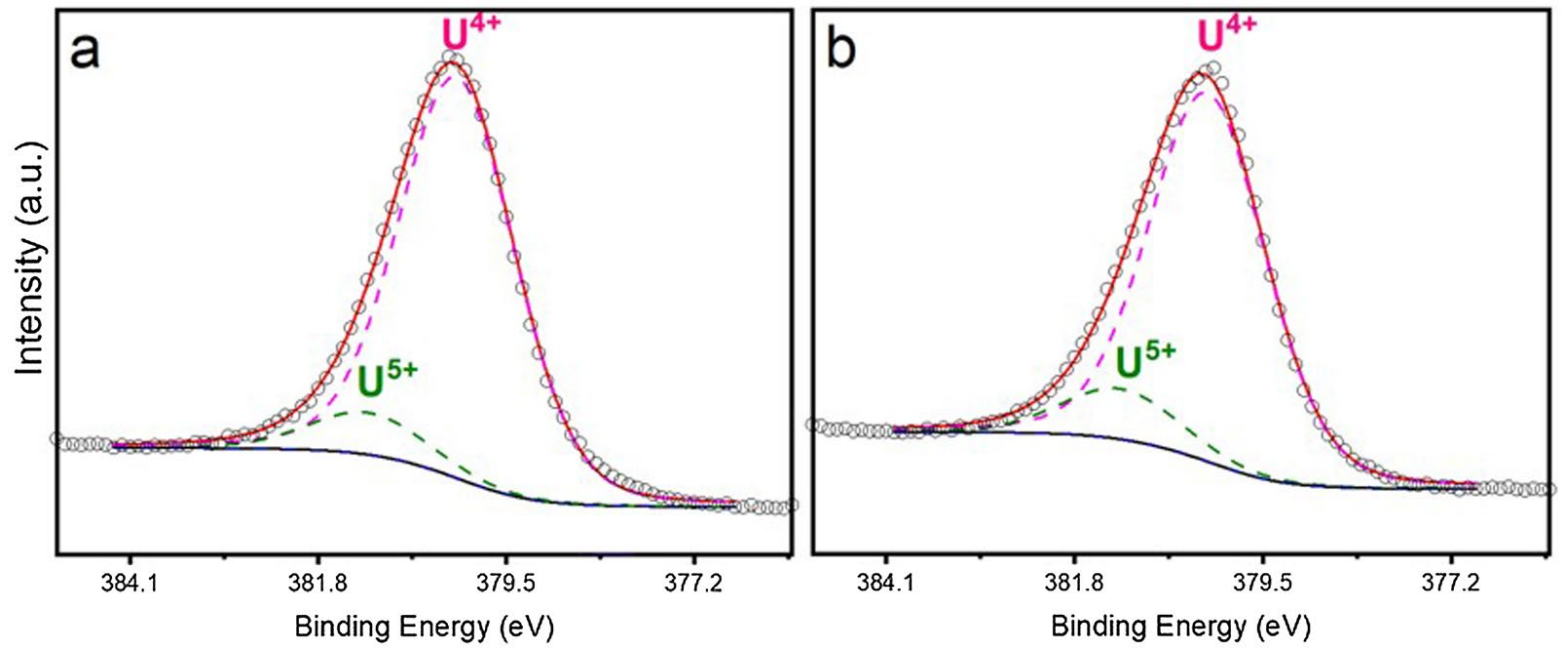
Deconvolution of the U *4f*_7/2_ photoelectron peaks for (**a**) nominal Ca_1.00_U_0.50_Zr_0.20_Ti_2.30_O_7_ and (**b**) nominal Ca_0.96_U_0.72_Zr_0.17_Ti_2.15_O_7_. The dashed lines are the fitting curves of U^4+^ and U^5+^ components. The dotted line and the solid line are the measured data and fitted contributions, respectively. The solid line at the bottom is baseline for the curve fitting.

The bulk average oxidation state of uranium in nominal Ca_1.00_U_0.50_Zr_0.20_Ti_2.30_O_7_ and Ca_0.96_U_0.72_Zr_0.17_Ti_2.15_O_7_ was further investigated using X-ray Photoelectron Spectroscopy (XPS). As shown in Fig. 5A, the spectra of both compositions presented two main peaks, U *4f*_7/2_ and U *4f*_5/2_ (separated by *ca.* 11.0 eV, due to spin–orbit splitting) and two satellite peaks, *Sat.*_7/2_ and *Sat.*_5/2_. Fitting of *Sat.*_*5/2*_ was used to assess the contributing uranium oxidation states; for both compositions, the *Sat.*_*5/2*_ peak could be fitted by a majority U^4+^ contribution with a minor U^5+^ contribution, as shown in Fig. 5B; no contribution from U^6+^ was apparent. Similarly, deconvolution of the U *4f*_7/2_ peak for nominal Ca_1.00_U_0.50_Zr_0.20_Ti_2.30_O_7_ and Ca_0.96_U_0.72_Zr_0.17_Ti_2.15_O_7_ evidenced a majority contribution from U^4+^ and a minor contribution from U^5+^, as shown in Fig. 6; no U^6+^ contribution was evidenced. The positions of two major components, with separations of 0.9 eV (U^4+^—U^5+^ in the deconvolution of *U 4f*_7/2_), are in agreement with those observed in the literature for mixed valence in uraninite^28^. The fractions of U^4+^ and U^5+^, as determined by peak deconvolution of *U 4f*_7/2_, are reported in Table 3.

As can be seen, a greater proportion of U^4+^ speciation was found in nominal Ca_1.00_U_0.50_Zr_0.20_Ti_2.30_O_7_ (91% U^4+^, 9% U^5+^) whereas uranium in nominal Ca_0.96_U_0.72_Zr_0.17_Ti_2.15_O_7_ was marginally more oxidised (U^4+^ 88%, U^5+^ 12%). Overall, the average bulk oxidation states determined from U L_3_-edge XANES and U *4f*_7/2_ XPS are in good agreement, and evidence dominant U^4+^ speciation with a minor U^5+^ contribution of around 10%. These bulk analyses are consistent with the dominant U^4+^ oxidation state inferred from EDX analyses of the betafite phase, within which the uranium is overwhelmingly partitioned. Our estimation of the U^5+^ content determined from XPS is based on curve fitting, assuming intrinsically symmetrical U *4f*_7/2_ lines. In reality these lines are slightly asymmetrical (multiplets) and the Shirley background is also only an approximation. These assumptions are expected to result in a small overestimation of the U^5+^ content. We can therefore conclude that the uranium speciation in the betafite phases is primarily U^4+^, with a U^5+^ contribution of no more than 10%.

Subsequent to this study, Blackburn et al*.* investigated the zirconolite solid solution CaZr_1-x_Th_x_Ti_2_O_7_, and discovered the formation of a new betafite phase, for x > 0.4; a single phase was produced for x = 0.6, with a determined composition of Ca_1.00(2)_Zr_0.33(2)_Th_0.54(1)_Ti_2.13(2)_O_7_^29^. The radius ratio of this phase is r_A_ / r_B_ = 1.70, within the pyrochlore stability field, and identical to that of the betafite phases designed and reported here. In contrast, McCauley and Hummel, reported synthesis of the end member composition CaThTi_2_O_7_, to be unsuccessful^30^. Indeed, this is consistent with a radius ratio, r_A_/r_B_ = 1.79, outside of the pyrochlore stability field. Therefore, Ca_1.00(2)_Zr_0.33(2)_Th_0.54(1)_Ti_2.13(2)_O_7_ may also be considered an example of a pyrochlore structure stabilised by Ti excess on the B-site and partial occupancy of the A-site. This example, and those reported herein, point to a wider family of actinide pyrochlores that could be stabilised by application of the underlying crystal chemical principle applied here.

## Conclusion

Novel U^4+^ dominant and titanium excess betafite phases, Ca_1.15(5)_U_0.56(4)_Zr_0.17(2)_Ti_2.19(2)_O_7_ and Ca_1.10(4)_U_0.68(4)_Zr_0.15(3)_Ti_2.12(2)_O_7_, were successfully synthesised in high yield (85 – 95 wt%), by application of the crystal chemical design principle of targeting excess B-site cations to the A-site in the pyrochlore structure. This design strategy enabled the radius ratio to be effectively tuned into the pyrochlore stability field, and the synthesis of U^4+^ betafite ceramics in high yield, and with high relative density (> 99% theoretical), for the first time. U L_3_-edge XANES and U *4f*_7/2_ and U *4f*_5/2_ XPS data evidenced U^4+^ as the dominant speciation, consistent with EDX determined compositions. Reconsideration of the recently reported thorium betafite phase, Ca_1.00(2)_Zr_0.33(2)_Th_0.54(1)_Ti_2.13(2)_O_7_ established that this compound is also effectively stabilised by the same crystal chemical design principle applied here. More broadly, this example, and the novel betafite phases designed and reported herein, point to a wider family of actinide pyrochlores that could be stabilised by application of same crystal chemical principle. The observed incorporation of Fe within Ca_0.90(5)_U_0.71(5)_Zr_0.15(2)_Ti_1.97(3)_Fe_0.28(6)_O_7_ demonstrates a further degree of chemical flexibility which could be exploited in terms of this crystal chemical design principle, with partial Ti^4+^ occupancy of the pyrochlore A-site facilitated by co-substitution on the B-site of a suitable cation.

## Methods

### Caution

Uranium is an alpha emitter. Manipulations, synthesis and characterisation were performed in a materials radiochemistry laboratory in a controlled area, using HEPA filtered fume hoods and a dedicated glove box, following risk assessments and monitoring procedures^31^.

Betafite ceramics were produced by solid-state reaction – sintering between stoichiometric quantities of CaTiO_3_ (Sigma-Aldrich, purity ≥ 99% trace metals basis), ZrO_2_ (Sigma-Aldrich, purity ≥ 99%) and TiO_2_ (Sigma-Aldrich, purity ≥ 99%) and UO_2_ (purity > 99%). UO_2_ with a small particle size of 1 μm was selected as a reagent, given previous suggestion that pyrochlore synthesis may be kinetically hindered by the use of UO_2_^16,18^. The target betafite compositions were Ca_1.00_U_0.50_Zr_0.20_Ti_2.30_O_7_ and Ca_0.96_U_0.72_Zr_0.17_Ti_2.15_O_7_, as discussed in the Introduction section. The mixture of reagents was ball milled for 16 h in high-density-polyethylene pots containing calcium stabilised zirconia media and isopropanol as a carrier fluid. The media were separated from the milled slurry and dried overnight at 90 °C. The master batch of Ca_0.96_U_0.72_Zr_0.17_Ti_2.15_O_7_ precursor was divided into three parts; 10 wt% of metallic Fe (Acros Organics, purity ≥ 99%) or Ni (Acros Organics, purity ≥ 99.9%) was added to one part of the precursor, by mixing in a mortar and pestle. These compositions were fabricated to investigate the potential for metallic Fe and Ni to maintain U^4+^ by scavenging trace oxygen. Batched material was uniaxially pressed in a 10 mm steel die under uniaxial pressure of 180 MPa and sintered at 1320 °C for 2 h, with a ramp rate of 5 °C·min^−1^, in flowing high purity nitrogen (250 mL·min^−1^).

The density of the sintered ceramics was measured based on Archimedes displacement method. For phase analysis, sintered ceramics were sectioned using a diamond saw and a small segment was ground to a fine powder in a mortar and pestle. Examination of the phase assemblage was performed by powder X-ray diffraction (PXRD; D2 Phaser, Bruker, Karlsruhe, Germany) with a Cu *K*_α_ source, Ni *K*_*β*_ filter operating voltage of 30 kV and current of 10 mA. Quantitative phase analysis (QPA) was performed by Rietveld refinement using the GSAS software package and the ExpGUI interface^32^. The microstructure of sintered pellets was examined by Scanning Electron Microscopy (SEM) in backscattered electron mode using a Hitachi TM3030 microscope coupled with a Bruker Quantax 70 EDX system. Samples were prepared for analysis by polishing sections of ceramic to a 0.25 µm finish using SiC paper and progressively finer diamond pastes. Semi-quantitative compositions were acquired by Energy Dispersive X-ray spectroscopy (EDX) based on 10 EDX data points; a stoichiometry of 7 O atoms per formula unit was assumed, given the low accuracy of EDX to light element determination.

Average uranium oxidation states were determined by analysis of U L_3_-edge X-ray absorption near edge structure (XANES). The ceramic products and reference compounds for XANES measurement were prepared by homogenously mixing powder specimens with polyethylene glycol and uniaxially pressing to form 13 mm diameter pellets of approximately one absorption length. XANES data were acquired on Beamline B18 at Diamond Light Source (DLS; Oxford, UK). The beamline configuration comprised a water cooled vertically collimated Si mirror, a double crystal Si(111) monochromator, a double toroidal focusing mirror, and harmonic rejection mirrors. Uranium L_3_-edge XANES spectra were recorded in transmission mode between 17,000 and 17,410 eV. To improve data quality, the beam spot size was defocused to ca. 1.0 mm and multiple scans were acquired and averaged. Data reduction and linear combination fitting were performed using the Athena software package^25^.

X-ray photoelectron spectroscopy (XPS) data of uranium were recorded at room temperature using a SPECS Phoibos 150 hemispherical analyser, using monochromated X-rays (SPECS, microfocus source, 15 kV, 50 W, spot size: 0.3 mm). Samples were glued with 2-component epoxy glue (Dynaloy 325) and stored under vacuum at room temperature for 7 days to allow cure finishing and avoid surface oxidation. Samples were scraped by a diamond file under vacuum (1 × 10^−7^ mbar) to produce bulk representative surfaces. The energy scale for XPS was calibrated with the Au *4f*_7/2_ (84.0 eV) and Cu *2p*_3/2_ (932.7 eV) emissions. The vacuum in the photoemission chamber were 1.2 × 10^−10^ mbar. Charge compensation was performed by a flood gun (1 eV, 15 mA). The obtained spectra were deconvoluted using Gaussian function and the baseline subtracted with a Shirley function. The location and full-width at half-maximum (FWHM) of the components were allowed to vary freely, but the width of the components was set to be equal in each fit.

## Supplementary Information

Supplementary Information.

## Data Availability

The datasets generated during and/or analysed during the current study are available from the corresponding author on reasonable request.
