# Supplementary material for: Crystal chemical design, synthesis and characterisation of U(IV)-dominant betafite phases for actinide immobilisation
Source: Sci Rep. 2023 Jun 26;13:10328. doi: 10.1038/s41598-023-36571-w (PMC10293183; doi:10.1038/s41598-023-36571-w)
Supplement: Supplementary file 1 — Supplementary Information. [file 41598_2023_36571_MOESM1_ESM.docx]

**Supplementary material for:**

**Crystal chemical design, synthesis and characterisation of U(IV)-dominant betafite phases for actinide immobilisation**

Shi-Kuan Sun,^1,2^ Lucy M. Mottram,^1^ Thomas Gouder,^3^ Martin C. Stennett,^1^
Neil C. Hyatt,^1,4,5^ Claire L. Corkhill.^1,5^*

1. Immobilisation Science Laboratory, Department of Materials Science and Engineering, University of Sheffield, Sheffield S1 3JD, United Kingdom

2. School of Material Science and Energy Engineering, Foshan University, Foshan, Guangdong 528000, China

3. European Commission, Joint Research Centre (JRC), Postfach 2340, D-76125 Karlsruhe, Germany.

4. School of Mechanical and Materials Engineering, Washington State University,

Pullman, WA 99164, USA.

5. School of Earth Sciences, University of Bristol, Bristol BS8 1RJ, UK.

* Corresponding author. Email address: c.corkhill@sheffield.ac.uk (C.L. Corkhill).


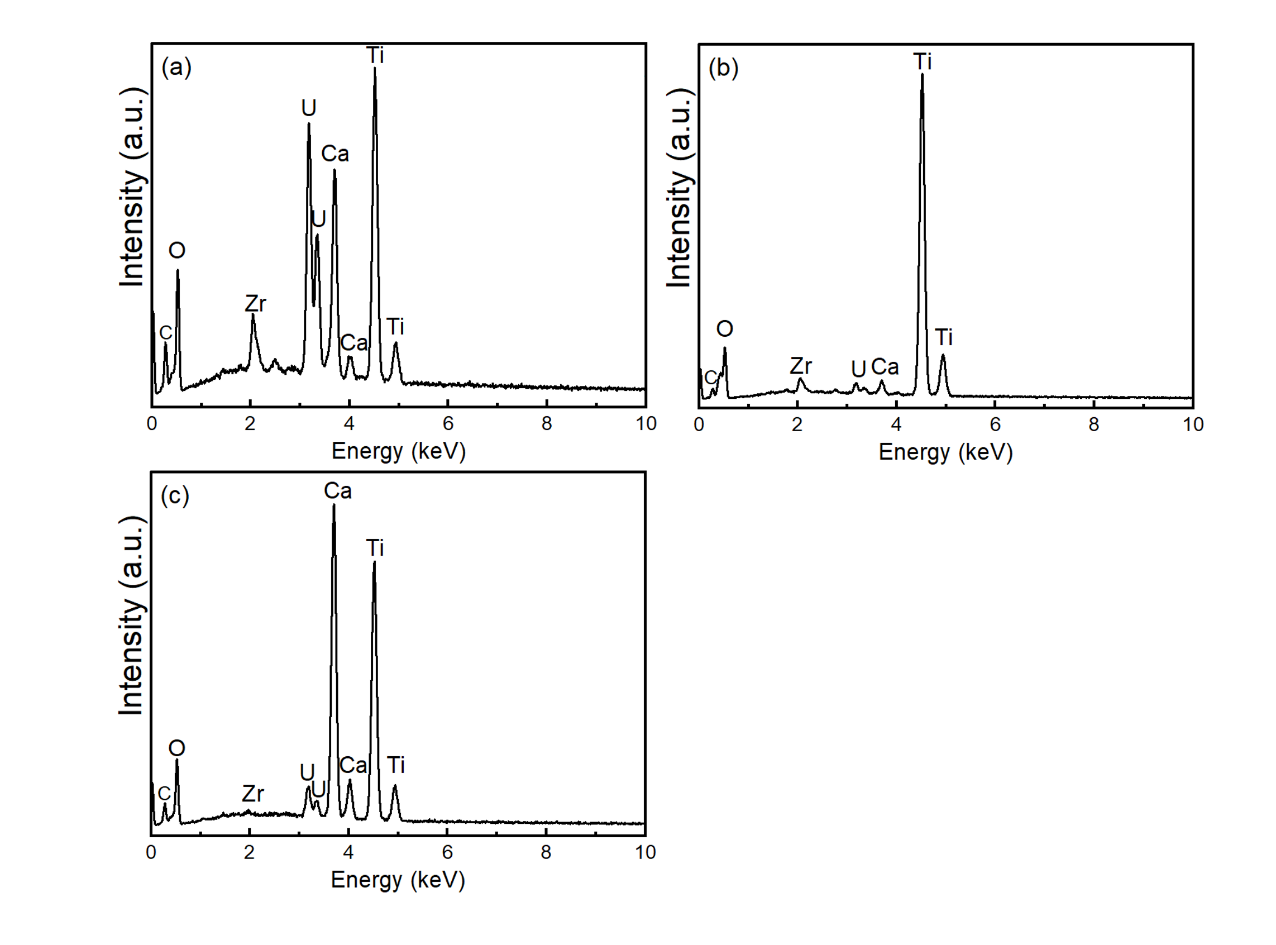


**Figure S1.** EDX spectra of (a) CaUTi_2_O_7_, (b) TiO_2_ and (c) CaTiO_3_ phases for nominal Ca_1.00_U_0.50_Zr_0.20_Ti_2.30_O_7_ composition. EDX determined compositions are reported in **Table 1** and **Table S1**.


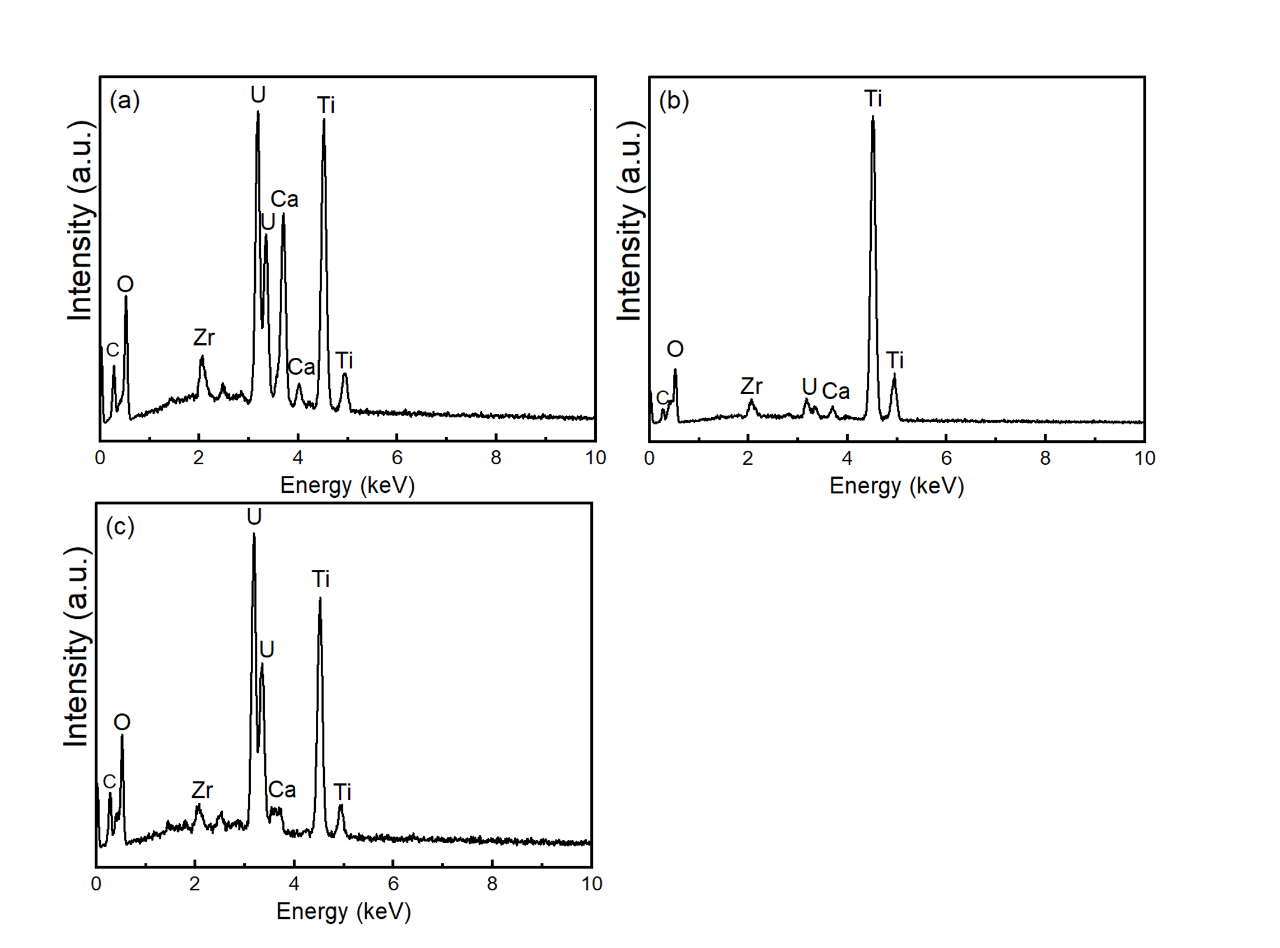


**Figure S2.** EDX spectra of (a) CaUTi_2_O_7_, (b) TiO_2_ and (c) UTi_2_O_6_ phases for nominal Ca_0.96_U_0.72_Zr_0.17_Ti_2.15_O_7_ composition. EDX determined compositions are reported in **Table 1** and **Table S1**.


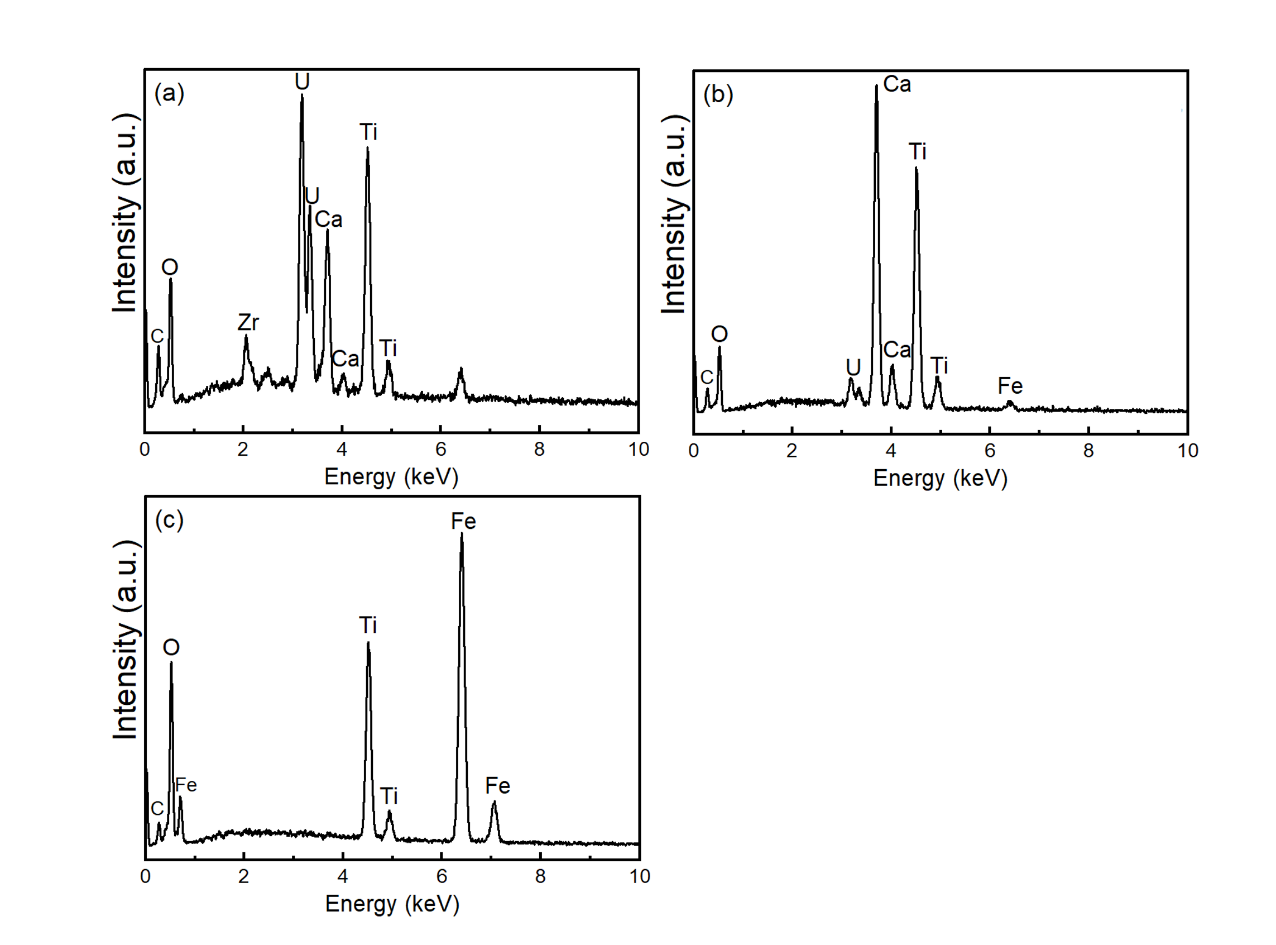


**Figure S3.** EDX spectra of (a) CaUTi_2_O_7_, (b) CaTiO_3_ and (c) Fe_2_TiO_4_ phases for 10wt% Fe addition to nominal Ca_0.96_U_0.72_Zr_0.17_Ti_2.15_O_7_ composition. EDX determined compositions are reported in **Table 1** and **Table S1**.


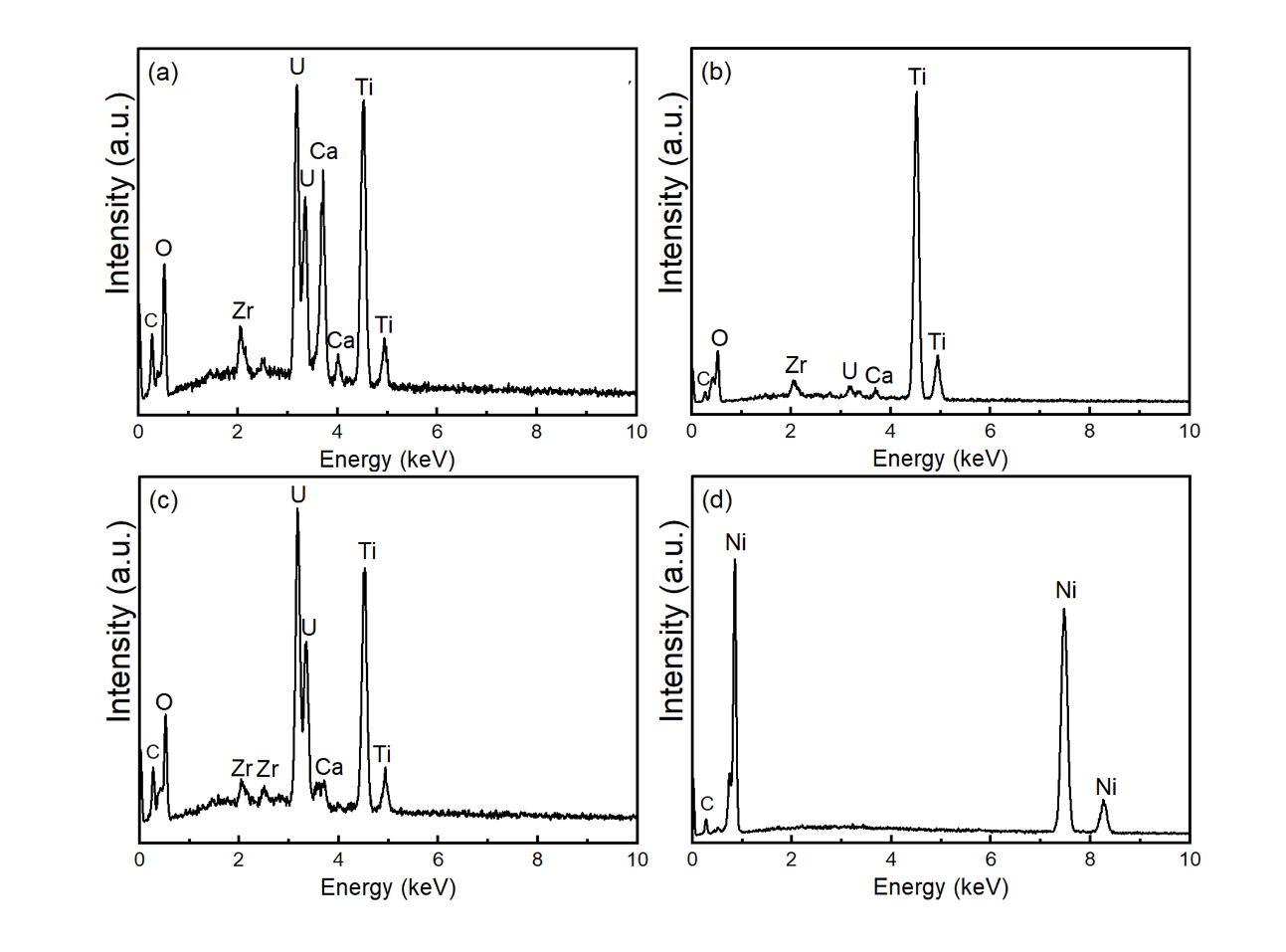


**Figure S4.** EDX spectra of (a) CaUTi_2_O_7_, (b) TiO_2_, (c) UTi_2_O_6_ and (d) Ni phases for 10wt% Ni addition to nominal Ca_0.96_U_0.72_Zr_0.17_Ti_2.15_O_7_ composition. EDX determined compositions are reported in **Table 1** and **Table S1**.


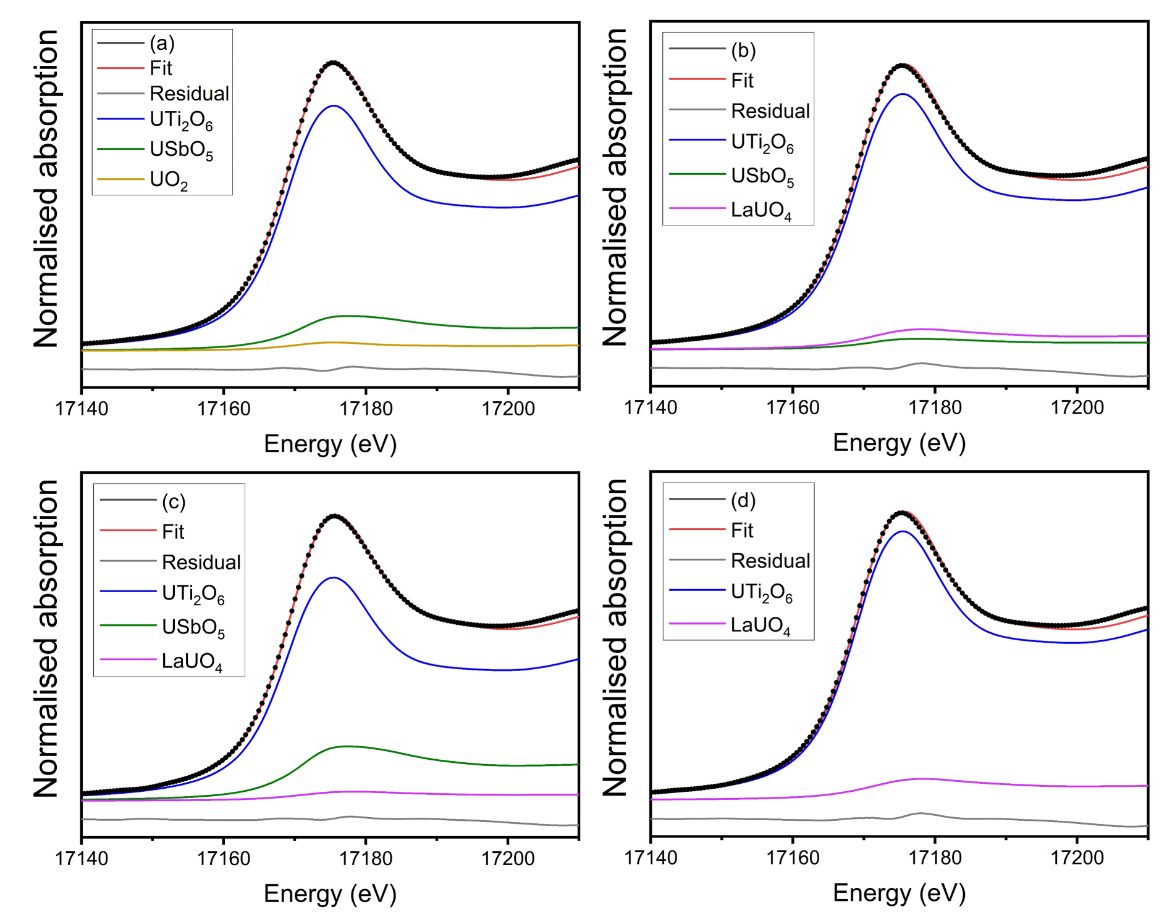


**Figure S5.** Combinatorial linear combination fitting of U L_3_-edge XANES data for (a) nominal Ca_1.00_U_0.50_Zr_0.20_Ti_2.30_O_7_, (b) nominal Ca_0.96_U_0.72_Zr_0.17_Ti_2.15_O_7_, and (c) 10wt% Fe addition to nominal Ca_0.96_U_0.72_Zr_0.17_Ti_2.15_O_7_ composition (d) 10wt% Ni addition to nominal Ca_0.96_U_0.72_Zr_0.17_Ti_2.15_O_7_ composition. Measured data shown as solid black points, linear combination fit shown as red solid line, residual difference shown as solid grey line; fitted components of reference compounds shown as solid coloured lines, as labelled.

**Table S1.** The composition of the secondary phases as determined by EDX in the betafite ceramics.

| Nominal Composition | Secondary phases | Secondary phase composition |
| --- | --- | --- |
| Ca_1.00_U_0.50_Zr_0.20_Ti_2.30_O_7_ | TiO_2_ | Ti_0.91(4)_O_2_ |
|  | CaTiO_3_ | Ca_0.87(13)_U_0.05(3)_Ti_1.08(12)_O_3_ |
| Ca_0.96_U_0.72_Zr_0.17_Ti_2.15_O_7_ | TiO_2_ | Ti_0.95(5)_O_2_ |
|  | UTi_2_O_6_ | U_0.78(7)_Ca_0.14(7)_Ti_1.99(8)_O_6_ |
| 10wt% Fe + Ca_0.96_U_0.72_Zr_0.17_Ti_2.15_O_7_ | CaTiO_3_ | Ca_0.91(2)_Ti_0.99(2)_O_3_ |
|  | Fe_2_TiO_4_ | Fe_2.16(8)_Ti_0.79(6)_O_4_ |
| 10wt% Ni + Ca_0.96_U_0.72_Zr_0.17_Ti_2.15_O_7_ | Ni | Ni |
|  | UTi_2_O_6_ | U_0.74(11)_Ca_0.10(5)_Zr_0.19(17)_Ti_1.96(16)_O_6_ |
|  | TiO_2_ | Ti_0.95(6)_O_2_ |

**Table S2**. Measured and estimated relative density of betafite ceramics.

| Nominal composition | Measured density (g·cm^-3^) | Estimated relative density  (% theoretical) |
| --- | --- | --- |
| Ca_1.00_U_0.50_Zr_0.20_Ti_2.30_O_7_ | 4.98 ± 0.01 | 99.5 |
| Ca_0.96_U_0.72_Zr_0.17_Ti_2.15_O_7_ | 5.38 ± 0.01 | 98.7 |
| Ca_0.96_U_0.72_Zr_0.17_Ti_2.15_O_7_ + 10wt% Fe | 4.40 ± 0.01 | 86.9 |
| Ca_0.96_U_0.72_Zr_0.17_Ti_2.15_O_7_ + 10wt% Ni | 5.56 ± 0.01 | 95.5 |
